# Associations between serum vitamin D and the risk of female reproductive tumors: A meta-analysis with trial sequential analysis

**DOI:** 10.1097/MD.0000000000010360

**Published:** 2018-04-13

**Authors:** Lina Yan, Yun Gu, Ting Luan, Miao Miao, Lisha Jiang, Yu Liu, Ping Li, Xin Zeng

**Affiliations:** a The Affiliated Obstetrics and Gynecology Hospital of Nanjing Medical University, Nanjing Maternity and Child Health Care Hospital, Nanjing; b Department of Obstetrics and Gynecology, The Second Affiliated Hospital of Medical University of Anhui, Hefei, China.

**Keywords:** female reproductive tumors, meta-analysis, serum vitamin D deficiency, trial sequential analysis

## Abstract

**Background:**

Female reproductive tumors are common with high morbidity and mortality worldwide; however, the association between gynecological tumors and serum vitamin D is controversial. The aim of this meta-analysis was to evaluate the relationship between insufficiency of serum vitamin D and the occurrence of benign and malignant gynecological tumors.

**Methods:**

Studies from inception to June 2017 were searched in the electronic databases: National Library of Medicine (PubMed), Web of Science (Clerivate), and Cochrane Database of Systematic Reviews (Cochrane Library, CDSR) by 2 investigators independently. Odds ratios (ORs) with 95% confidence intervals (CIs) were calculated using a random-effects model. STATA 12.0 Software and Trial Sequential Analysis (TSA) software were applied for data analyses.

**Results:**

Overall, 8 studies (including 2391 patients and 5798 patients with and without female reproductive tumors, respectively) were eligible for the present meta-analysis. In the subsequent meta-analysis, the occurrence of vitamin D deficiency in the case and control groups were 52.36% and 48.70%, respectively; women with female reproductive benign and malignant tumors were 55.57% and 50.59%, respectively. Although, no conclusive association was found between vitamin D deficiency and female reproductive tumors (OR, 1.05; 95% CI, 0.85–1.31); vitamin D deficiency may be a risk factor of malignant female reproductive neoplasm, as shown by the pooled OR (95% CI):1.17 (1.02–1.33). Furthermore, based on the OR values, association of vitamin D insufficiency with disease type, study location, number of patients, and methods for detecting CLA was observed. Similar results in the sensitivity analysis were observed. TSA showed that the cumulative *Z*-curve crossed the traditional boundary line, rather than crossing the trial sequential monitoring boundary. However, the cumulative information failed to reach the required information size.

**Conclusions:**

Currently, vitamin D deficiency appears to be a common issue in females, and there may be an urgent need to improve the level of vitamin D. Furthermore, vitamin D deficiency may be a non-negligible risk factor of malignant female reproductive neoplasm. Undoubtedly, more trials are required in the future according to TSA.

## Abstract 2

Supplemental Digital Content is available in the text

## 1 Introduction

Tumors of the reproductive system are one of the leading causes of morbidity and mortality among women worldwide, mainly occurring in the female uterus, cervix, ovaries, vulva, and vagina. The most common female reproductive cancer is endometrial cancer, accounting for nearly 7% of all new cancer cases detected among women in the United States in 2017.^[1]^ The second most common cancer among women worldwide is the cervix cancer, which is a serious female health problem accounting for two-thirds of gynecological cancers.^[2]^ Ovarian cancer is the seventh most common cancer and the eighth cause of death worldwide, with a high incidence in Europe and North America especially; and is often diagnosed as an advanced disease.^[3,4]^ Cancers of the vulva and vagina are rare with respective proportions of 3% and 2% or less in gynecological cancers.^[2,5]^ Nevertheless, regardless of these malignant tumors in females, some benign gynecological tumors also have negative influences on women's lives. Taking uterine fibroids as an example, it potentially influences women's health because it results in abnormal uterine bleeding, urinary dysfunction, constipation, pain, infertility, miscarriage, and some pregnancy complications.^[6]^ Therapies for the female reproductive tumors include surgery, chemotherapy, radiation therapy, and expectant treatments at present.^[7]^ Patients undergoing these treatments modalities bear higher expenses than expected. Expected cost covers medication, transportation, supplies and equipment, alternative therapies, and loss of income.^[8]^ In this sense, it is urgently required that the risk factors of female reproductive tumors should be studied to prevent their occurrence or reduce their incidence.

Combined effect of many factors, such as lifestyle behaviors, age, family history, environmental influences, diet, and nutrition may cause female reproductive tumors.^[9]^ In recent years, increasing numbers of researches have focused on the intake of specific nutrients or vitamins, and the relationship between vitamins and reduced incidence of cancers.^[10]^ As a compelling evidence, Gorham et al^[11]^ found that vitamin D could reduce the incidence of colorectal cancer. Meanwhile, Stearns and Visvanathan^[12]^ claimed that inadequate vitamin D could enhance the occurrence and mortality of breast cancer. Furthermore, low levels of vitamin D were found to be significantly associated with high risk of ovarian cancer among overweight and obese women.^[10]^ More recent researches concerning the relationship between vitamin D levels and female reproductive tumors have become increasingly necessary worldwide.

There are 2 forms of vitamin D (vitamin D_3_ and vitamin D_2_), a liposoluble vitamin highly abundant in foods, including fish, liver, milk, eggs, and waxy-leaf nightshade (*Solanum glaucophyllum*).^[13]^ Vitamin D_3_ is formed in the skin through solar ultraviolet (UV)-B radiation exposure, while vitamin D_2_ is endogenically synthesized from irradiation of ergosterol. The active form of vitamin D is 25-hydroxyvitamin D_3,_ which results from itshydroxylation on carbon 25 in the liver to form 25-hydroxyvitamin D, then on carbon 1 in the kidney to form 1,25-dihydroxyvitamin D.^[14]^ As reported in existing studies, vitamin D has different forms, such as serum vitamin D, tissue vitamin D, and vitamin D gene receptor. Based on the clinical significance, serum 25-hydroxyvitamin D level is widely recognized as a biomarker in determining the effect of short-term vitamin D status.^[1]^

Serum vitamin D deficiency and insufficiency is highly prevalent worldwide, and has gradually become a global public health concern.^[15,16]^ In the past decades, significant relationships have been found between vitamin D deficiency and several physiologic systems, such as the formation of bone, prevention of several degenerative diseases, and anticancer ability.^[10,17]^ As earlier mentioned, raising the level of serum vitamin D could reduce the incidence of certain cancers. More importantly, conflicting results were found in studies on vitamin D levels and female reproductive tumors. Previously, McCullough et al^[18]^ came to the conclusion that no relationship existed between vitamin D and endometrial cancer. However, circulating vitamin D was found inversely associated with the incidence of ovarian cancer.^[19]^ In view of these controversial facts on the impact of vitamin D on the female reproductive tumor incidence, comprehensive researches are required, as prior studies were usually based on single female reproductive tumor types. It was also inconclusive whether vitamin D supplement was beneficial for reducing the incidence of female reproductive tumors or improved physical health of women.

Thus, a comprehensive meta-analysis was conducted here for further evaluation of the relationship between serum vitamin D levels and female reproductive tumors, thereby providing references for the early intervention of gynecological cancers.

## 2 Materials and methods

### 2.1 Search strategy

Our meta-analysis was conducted in accordance with Preferred Reporting Items for Systematic Reviews and Meta-Analyses (PRISMA) guidelines.^[20]^ A comprehensive search of major electronic databases was conducted for literature on serum vitamin D and female reproductive tumors up to June 2017. The following databases were covered: National Library of Medicine (PubMed), Web of Science (Clerivate), and Cochrane Database of Systematic Reviews (Cochrane Library, CDSR). The search utilized the keywords “vitamin D,” “uterine neoplasm,” “endometrial neoplasm,” “uterine cervical neoplasm,” “ovarian neoplasm,” “oviduct neoplasm,” “vaginal neoplasm,” and “vulvar neoplasm.” More details regarding the terms are provided in the Supplemental Digital Content. Furthermore, references of relevant articles were also analyzed to avoid missing eligible articles.

### 2.2 Inclusion and exclusion criteria

Two investigators independently searched and reviewed articles for eligibility via the following inclusion criteria: all studies focusing on patients with female reproductive benign and malignant tumors without limitations on age; studies published in English; in accordance with the Endocrine Society Guidelines, vitamin D deficiency was defined as serum 25(OH)D ≤20 ng/mL (≤50 nmol/L); and adequate data for extracting or calculating. Furthermore, it should be mentioned that studies focusing on endometriosis were also included. Recently, reports of endometriosis-associated benign or malignant neoplasm are increasing.^[21–23]^ Hence, in a sense, endometriosis could be thought of as a tumor.

Studies were excluded based on the following criteria: the level of vitamin D were divided by different criterion or data being incomplete; duplicate publication of articles; obscurely reported outcomes, or lack of control groups; and animal studies, case reports, basic researches, meeting summary and general overviews.

### 2.3 Data extraction and quality assessment

The data extracted from the studies included: last name of the first author, publication year, study region, tumor types, age, the level of serum vitamin D, methods of vitamin D detection, and number of cases and controls, the number of cases and controls with vitamin D deficiency.

The quality of the studies included was assessed using the Newcastle–Ottawa scale (NOS).^[24]^ The quality was evaluated using the following items: patient selection, comparability, and assessment of outcome. The total score was 9, with the definition that 0 to 4 meant low quality researches, while 5 to 9 meant high quality.^[25]^ The above process was performed by 2 independent investigators and a third investigator was consulted when there was any uncertainty. Disagreements were resolved by consensus. Furthermore, our meta-analysis was based on secondary data; thus, the ethical approval or patient consent was not necessary.

### 2.4 Statistical analysis

Meta-analysis was performed using STATA version 12.0 (StataCorp, College Station, TX), while association between vitamin D deficiency and female reproductive tumors was evaluated using odds ratio (OR) and 95% confidence interval (CI). In addition, heterogeneity was assessed using *Q* test and *I*^*2*^ test. The fixed effect model was used when there was no heterogeneity as indicated by *P* value > .10 and *I*^*2*^ < 50%, or else, the random effect model was applied. Efficiency and sensibility of Begg and Egger test were too poor when studies included were < 20. To assess for publication bias, visual inspection of the funnel plots was done and an asymmetric plot indicated potential publication bias.^[25]^

Furthermore, with smaller numbers of studies and patients, random error would increase and meta-analyses might result in type-I error.^[26]^ Thus, to determine whether cumulative sample size was powered for the obtained effect and to avoid random error, trial sequential analysis (TSA) was applied using TSA version 0.9.5.5 beta (TSA 2016; www.ctu.dk/tsa). Moreover, it provided adjusted thresholds for both statistical significance and futility according the quantified strength of the evidence and the impact of multiplicity.^[27]^ We constructed *Z*-curves for both primary and secondary outcomes, and alpha conventional threshold for significance testing was set at 5%. The expected intervention effect may be achieved and no further trials required when the cumulative *Z*-curve crosses the trial sequential monitoring boundary or enters the futility area. If the *Z*-curve does not cross any of the boundaries and the required information sample size has not been reached, more trials should be included. In instances when the cumulative *Z*-curve exceeds the estimated information size but does not cross the traditional monitoring boundary, the negative conclusion is sufficient and no further trial is required.^[28]^

## 3 Results

### 3.1 Characteristics of the included studies

**Figure 1:**
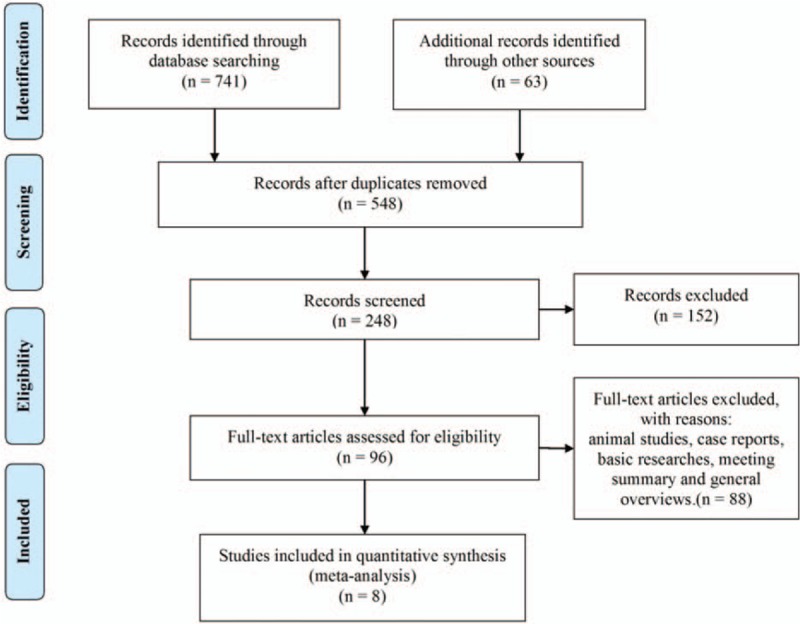
The flow diagram of studies selection.

According to the inclusion and exclusion criteria, eight case–control studies published from 2009 to 2016 were included. Among them, 4 articles reported that vitamin D were associated with benign gynecological tumors and 4 with malignancies.^[29–36]^ As shown in the study flow diagram in Figure 1, a total of 8189 patients (2391 cases and 5798 controls) were included. In addition, 8 articles were used in the pooled analysis. ORs and 95% CIs of 8189 patients were evaluated. Vitamin D deficiency was reported in 853 (55.57%) women with gynecological benign tumor and 1538 (50.59%) with malignancy. The descriptive characteristics are presented in Table 1 while the detailed quality assessment is shown in Table 2.

**Table 1:**
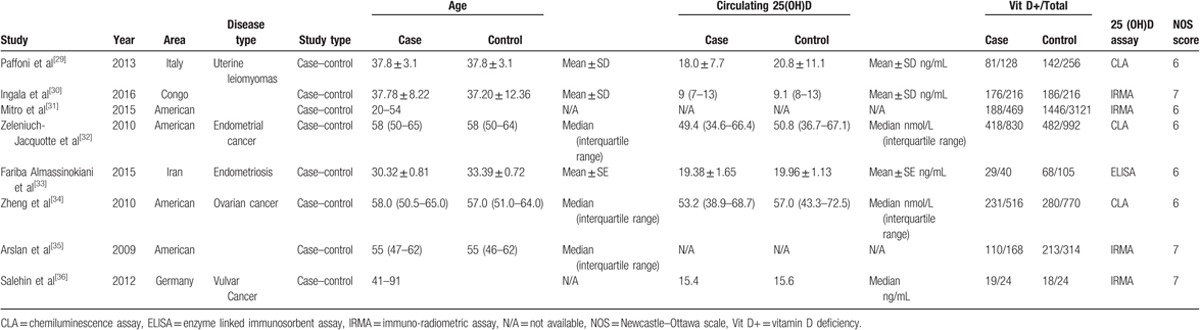
Characteristics of the included studies.

**Table 2:**
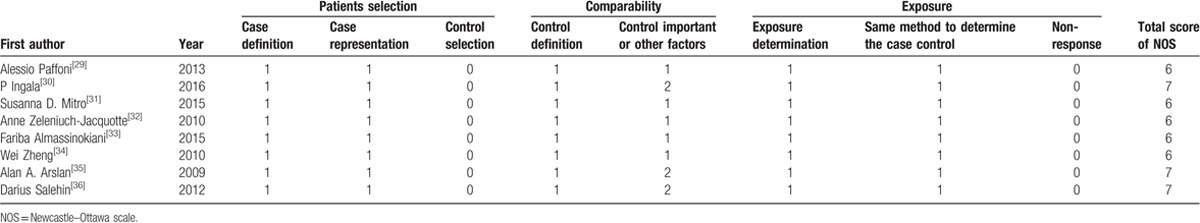
Quality of Newcastle-Ottawa scale (NOS).

### 3.2 Overall meta-analysis of vitamin D in female reproductive tumors

**Figure 2:**
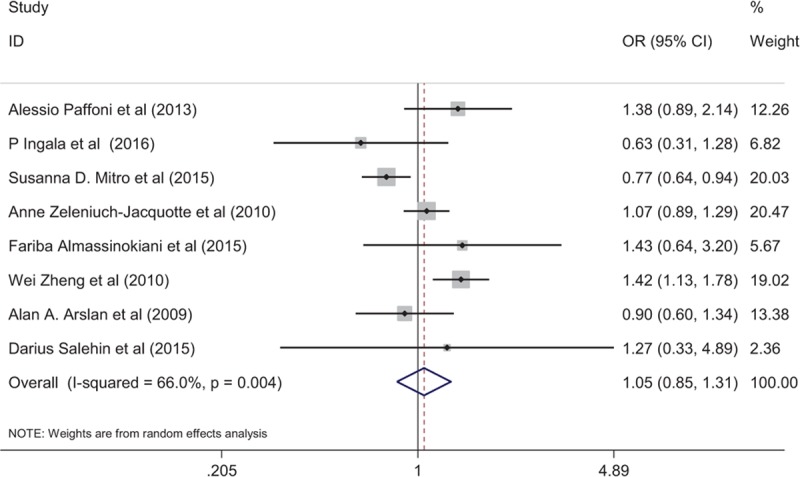
Forest plot shows the relation between vitamin D and female reproductive tumors.

Heterogeneity was found in the overall meta-analysis of all eligible studies, as *I*^*2*^ = 66.0% (*I*^*2*^ > 50%) and *P* = .004 (*P* < .1). Consequently, the random-effects model was used for the meta-analysis. The result of meta-analysis showed that a pooled OR (95% CI) for the association between vitamin D deficiency and the included female reproductive tumors was 1.05 (0.85–1.31), as shown by the forest plots (Fig. 2).

### 3.3 Subgroup analysis of vitamin D insufficiency

Subgroup analysis was then conducted by contraposing tumor types, publication year, study location, number of patients, and detection methods. From the subgroup analysis, a significant relationship was indicated between vitamin D insufficiency and the included female gynecological cancers and benign tumors (OR, 0.97; 95% CI, 0.66–1.42; *P* = .047), developed country (OR, 1.07; 95% CI, 0.85,1.36; *P* = .003), more than 1000 patients (OR, 1.05; 95% CI, 0.74–1.46; *P* = .000), but not with malignant tumors (OR, 1.17; 95% CI, 1.02–1.33; *P* = .236), publication year < 2015 (OR, 1.18; 95% CI, 0.99–1.44; *P* = .210) or ≥2015 (OR, 0.81; 95% CI, 0.60–1.09; *P* = .279), less than 1000 patients (OR, 1.05; 95% CI, 0.74–1.41; *P* = .331), method for detecting chemiluminescence assay (CLA) (OR, 1.25; 95% CI, 1.01–1.53; *P* = .146) and IRMA (OR, 0.79; 95% CI, 0.67–0.94; *P* = .728) (Table 3).

**Table 3:**
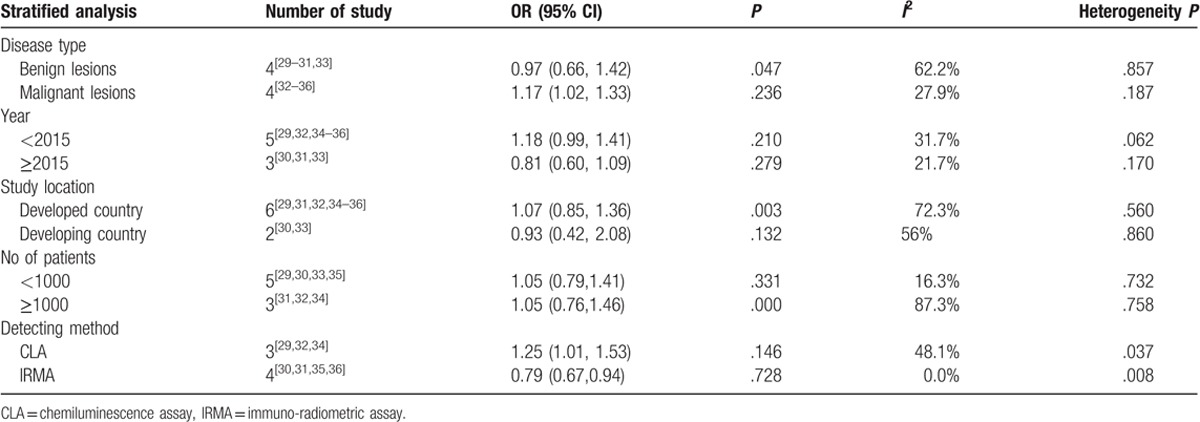
The subgroup analysis of vitamin D deficiency and female reproductive cancers.

### 3.4 Sensitivity analysis of vitamin D insufficiency

**Figure 3:**
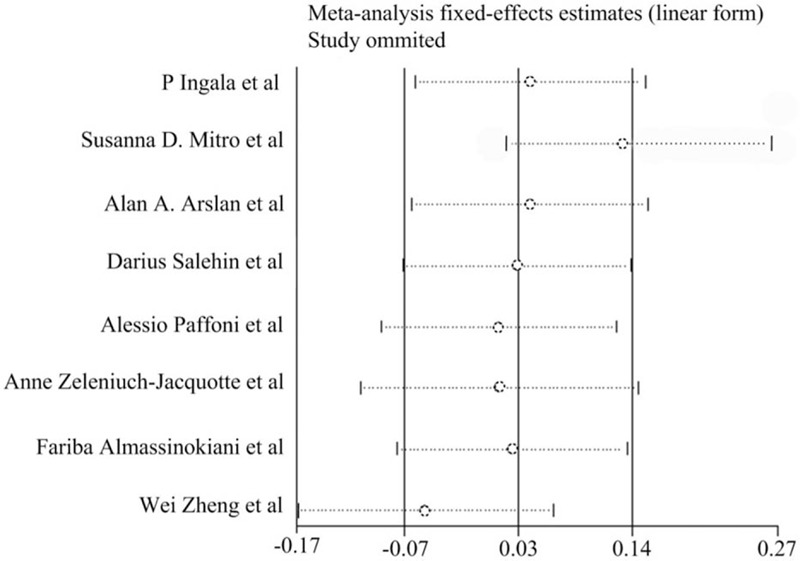
Sensitivity analysis about serum vitamin D insufficient of benign and malignant lesions in female reproductive system. The study of Mitro et al and Zheng et al may be the origin of heterogeneity. The detailed data was shown in supplement table.

A sensitivity analysis of serum vitamin D insufficiency with benign and malignant gynecological tumors was conducted by eliminating each study included in the meta-analysis individually. However, no statistically significant changes were found on conclusion as shown in Fig. 3.

### 3.5 Publication bias

**Figure 4:**
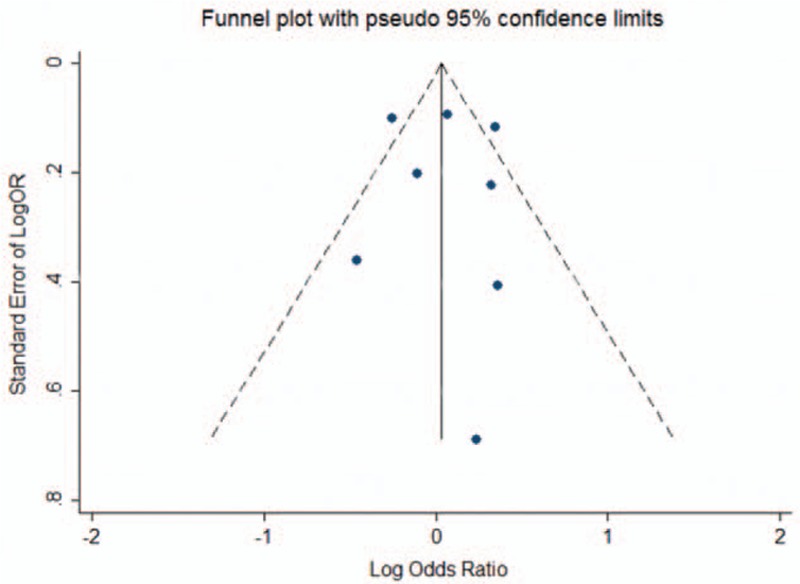
The funnel plot about serum vitamin D of benign and malignant lesions in female reproductive system.

Potential publication bias was assessed by visual inspection of the funnel plots (Fig. 4), and the symmetric plot suggested no evidence of publication bias.

### 3.6 Overall TSA of the studies included

**Figure 5:**
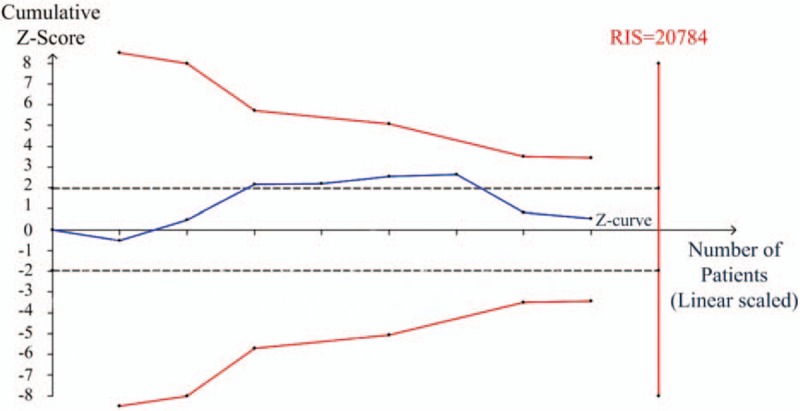
Trial sequential analysis (TSA) about serum vitamin D insufficient of benign and malignant lesions in female reproductive system. The solid blue line is the cumulative *Z*-curve. To the left, the red, inward-sloping, solid lines make up the trial sequential monitoring boundaries. To the right, the vertical red line represents required information size (RIS) of this meta-analysis. The black dashed lines represent the conventional statistical boundaries. TSA = trial sequential analysis.

Eight articles (8189 patients) were included, although 20784 participants were required according to TSA. As shown in Figure 5, the cumulative *Z*-curve (blue line) crosses the traditional boundary line rather than crossing the trial sequential monitoring boundary (red line), and the cumulative information failed to reach the required information size (RIS).

## 4 Discussion

Gynecological tumor is an important cause of death globally and accounted for approximately 10.35% of cancer-related deaths.^[37]^ Among the various risk factors of female reproductive tumors, vitamin D could not be ignored. Previously, there were studies stating that vitamin D deficiency could increase the risk of some specific gynecological tumors, such as ovarian cancer, uterine fibroids, and endometrial cancer.^[18]^ However, it remains controversial whether hypovitaminosis D is a consequence or factor predisposing to female reproductive tumors. Hence, the present meta-analysis as the latest study, pooled the data of 8189 patients to demonstrate the association between serum vitamin D and multiple gynecological tumors. Furthermore, detailed analysis was conducted based on our findings.

Contrary to findings in other researches, the results of our meta-analysis based on 8189 patients found no conclusive association between vitamin D deficiency and the risk of female reproductive tumors, and the pooled OR (95% CI) was 1.05 (0.85–1.31).^[11,12]^ Similar conclusions were also found on female reproductive benign tumors (OR: 0.97; 95% CI: 0.66–1.42). However, vitamin D deficiency may be a risk factor for malignant female reproductive neoplasm, as the pooled OR (95% CI) was 1.17 (1.02–1.33). Besides, the incidence of vitamin D deficiency was high in women with female reproductive benign tumor (55.57%) or malignant tumor (50.59%). The incidences of vitamin D deficiency were also high in the case (52.36%) groups and the control groups (48.70%). In other words, vitamin D deficiency may be anon-negligible regulator of tumor occurrence and progression. Thus, it is urgently necessary to improve the level of vitamin D among women. These findings have uncovered new insights for future functional studies on gynecologic neoplasm. This will also promote further development of effective prevention, diagnosis, and therapy for female reproductive neoplasms.

These results might be affected by tumor type, study location, detection methods, and number of patients according to our subgroup analysis, which is discussed below. As mentioned above, vitamin D deficiency was common in diseases with bone loss than in those with cardiovascular dysfunction, and this could increase the incidence of ovarian cancer rather than other cancer.^[17,19]^ In general, the risk of female reproductive tumors was different among various disease types when vitamin D was deficient. Moreover, there were researches claiming that residents of the northeastern United States and individuals with more skin pigmentation were at increased risk of vitamin D deficiency.^[38]^ A study included in our meta-analysis reported that insufficient vitamin D was associated with uterine leiomyoma in white but not black women, which may suggest different latitude and race could influence the risk of female reproductive tumors.^[6]^ Another influence deserving discussion is the sensitivity and specificity which are expected to be different with diverse detection methods. In the last 4 decades, the detection methods of vitamin D has undergone continuous change from the early competitive binding assays to immunoassay and liquid chromatography and currently mass spectrometry.^[39]^ With the improvement of the detection methods, the total detectable rate of vitamin D deficiency in female reproductive tumors may have risen, and this may influence the constituent ratios of female reproductive tumor risks. Although 8189 patients were included in our meta-analysis, the distribution of sample size in each article was unbalanced. Additionally, some included studies had a small sample size, which could undermine the reliability between vitamin D deficiency and the incidence of female reproductive tumors.^[40]^ In spite of the influence of the factors mentioned above, the results of our meta-analysis were reliable and meaningful, according to further analysis on sensitivity and bias.

The distinctiveness of the present meta-analysis was that we conducted a TSA, which could provide information on optimum sample size, and boundaries for estimating whether it was reliable or futile.^[41]^ It could also reduce the likelihood of false positive results arising from cumulative meta-analyses that involve multiple statistical tests. However, the limitation of TSA, which included heterogeneity, still existed in the study designs, study populations, and trial results, although the heterogeneity in the existing study results had already been incorporated into the calculations for the TSA.^[42]^

Another distinctiveness of the above meta-analysis was that an article on endometriosis was also included. According to epidemiologic, histopathologic and molecular data, endometriosis has features of tumor.^[43]^ Taniguchi^[44]^ reported that the prevalence of ovarian cancer was higher in women with endometriosis than the general population. Although endometriosis has malignant potentials, it may be more appropriate to classify it as a benign tumor. Therefore, articles on endometriosis with vitamin D deficiency were searched and those eligible were included.

Besides, some significant clinical findings were observed in our meta-analysis. Hypovitaminosis D was common both in the case and control groups, and in the female reproductive benign and malignant tumor group; thus, indicating a need to raise the level of vitamin D urgently. Similar to the fat-soluble secosteroid vitamin, vitamin D has pleiotropic functions in some clinical applications especially in regulating metabolism.^[45]^ Supplementation of calcium and vitamin D can regulate bone metabolism, and bone loss and also reduce the incidence of fractures. In the metabolism of immunocytes, Segaert et al^[46]^ found that corticosteroids and vitamin D analogs could lead to the disruption of the inflammatory feedback loop in anti-inflammatory and immunomodulatory of psoriasis. Although the causes of tumor were still unclear, increasing evidence suggested that metabolism disorder was significant in the occurrence and progression of tumor, and vitamin D may assume an important role for tumors by regulating metabolism.^[47]^ Thus, vitamin D was considered to have potential as an anti-cancer agent showing significant anti-tumor activity in vitro and in vivo in prostate, breast, colorectal, head/neck, and lung cancer.^[10]^ In other words, vitamin D supplementation could provide new insights for prevention or therapy in the precision medicine of female reproductive tumors, which could be beneficial for improving the prognosis and delaying the progression of tumors. In other words, it may enhance the efficacy of combination therapy in tumor. However, after long-term use of daily multivitamin, benefits were not found among populations with cardiovascular diseases, certain cancers or cognitive dysfunctions.^[48]^ Meanwhile, increasing studies indicated that high-dose vitamin supplementation could have some negative impacts, such as metabolic disorders, reduced fertility, fetal malformation or vitamin dependence syndrome, and may even increase all-cause mortality including cancer.^[49]^ Therefore, the mechanism of vitamin D in female reproductive tumors needs to be studied and verified thoroughly with more basic and clinical tests. In addition, the necessity and safety of vitamin D supplement also need further evaluation.

In spite of these findings, limitations of this study should be mentioned. Firstly, only 8 studies (8189 patients) were included and the TSA results indicated that the quantity of researched patients was insufficient. Secondly, studies in English alone were included, so selection bias might exist. Thirdly, there were limited data on factors of vitamin D deficiency, such as menstrual status, age, smoking and alcohol status which might have altered the level of vitamin D. Finally, although our meta-analysis was not registered, the procedure was conducted strictly following the rules of meta-analysis. Existence of bias was unavoidable in the present study and further studies aimed at researching the effect of vitamin D on female reproductive tumors with more comprehensive data are urgently required.

In conclusion, this is the latest meta-analysis demonstrating the association between serum vitamin D and gynecological tumors. Vitamin D deficiency may be common among females now and the level of vitamin D may need to be urgently improved. In addition, vitamin D deficiency may be a non-negligible risk factor for malignant female reproductive neoplasms. Undoubtedly, more basic and clinical tests are required to further explore whether vitamin D is a possible biomarker to predict the incidence of gynecological tumors, and whether vitamin D supplementation could enhance the prognosis and progression of female reproductive tumors.

## Author contributions

**Conceptualization:** L. Jiang, L. Yan, M. Miao, P. Li, T. Luan, X. Zeng, Y. Gu, Y. Liu.

**Data curation:** L. Yan, Y. Gu.

**Formal analysis:** L. Jiang, L. Yan, T. Luan, Y. Gu.

**Funding acquisition:** P. Li, X. Zeng.

**Investigation:** L. Yan, Y. Gu.

**Methodology:** L. Jiang, L. Yan, M. Miao, P. Li, T. Luan, Y. Gu, Y. Liu.

**Project administration:** X. Zeng.

**Resources:** L. Yan, Y. Gu.

**Software:** L. Yan, T. Luan.

**Supervision:** M. Miao, P. Li, X. Zeng, Y. Gu.

**Validation:** M. Miao, P. Li, X. Zeng, Y. Gu.

**Visualization:** M. Miao, P. Li, X. Zeng, Y. Gu.

**Writing – original draft:** L. Yan, Y. Gu.

**Writing – review & editing:** P. Li, X. Zeng, Y. Gu.

## Supplementary Material

Supplemental Digital Content
