# Associations between serum vitamin D and the risk of female reproductive tumors: A meta-analysis with trial sequential analysis: Erratum

**DOI:** 10.1097/MD.0000000000011431

**Published:** 2018-06-29

In the article, “Associations between serum vitamin D and the risk of female reproductive tumors: A meta-analysis with trial sequential analysis”,^[1]^ which appeared in Volume 97, Issue 15 of *Medicine*, Lina Yan should not be listed as a corresponding author.
