# Supplementary material for: Associations between serum vitamin D and the risk of female reproductive tumors: A meta-analysis with trial sequential analysis
Source: Medicine (Baltimore). 2018 Apr 13;97(15):e0360. doi: 10.1097/MD.0000000000010360 (PMC5908580; doi:10.1097/MD.0000000000010360)
Supplement: Supplemental Digital Content [file medi-97-e0360-s001.docx]

**Supplementary 1 Search strategy**

**Terms list**

**1) National Library of Medicine (PubMed): (Up to 31-** **June)**

#1 "Vitamin D" OR "Cholecalciferol" OR "Hydroxycholecalciferols" OR "Ergocalciferols" OR "25-Hydroxyvitamin D 2" OR "Dihydrotachysterol"

#2 "Uterus Neoplasm" OR "Uterus Carcinoma" OR "Uterus Cancer"

#3 "Endometrial Neoplasm" OR "Endometrial Carcinoma" OR "Endometrial Cancer"

#4 "Uterine Cervical Neoplasm" OR "Uterine Cervical Carcinoma" OR "Uterine Cervical Cancer"

#5 "Ovarian Neoplasm" OR "Ovarian Carcinoma" OR "Ovarian Cancer"

#6 "Oviduct Neoplasm" OR "Oviduct Carcinoma" OR "Oviduct Cancer"

#7 "Vaginal Neoplasm" OR "Vaginal Carcinoma" OR "Vaginal Cancer"

#8 "Ulvar Neoplasm" OR "Ulvar Carcinoma" OR "Ulvar Cancer"

#9 #2 OR #3 OR #4 OR #5 OR #6 OR #7 OR #8

#10 #1 AND #9

**2) Web of Science (Clerivate),**

#1 TS = ("Vitamin D" OR "Cholecalciferol" OR "Hydroxycholecalciferols" OR "Ergocalciferols" OR "25-Hydroxyvitamin D 2" OR "Dihydrotachysterol")

#2 TS = ("Uterus Neoplasm" OR "Uterus Carcinoma" OR "Uterus Cancer")

#3 TS = ("Endometrial Neoplasm" OR "Endometrial Carcinoma" OR "Endometrial Cancer")

#4 TS = ("Uterine Cervical Neoplasm" OR "Uterine Cervical Carcinoma" OR "Uterine Cervical Cancer")

#5 TS = ("Ovarian Neoplasm " OR "Ovarian Carcinoma" OR "Ovarian Cancer")

#6 TS = ("Oviduct Neoplasm" OR "Oviduct Carcinoma" OR "Oviduct Cancer")

#7 TS = ("Vaginal Neoplasm" OR "Vaginal Carcinoma" OR "Vaginal Cancer")

#8 TS = ("Ulvar Neoplasm" OR "Ulvar Carcinoma" OR "Ulvar Cancer")

#9 TS = (#2 OR #3 OR #4 OR #5 OR #6 OR #7 OR #8)

#10 TS = (#1 AND #9)

**3) Cochrane Database of Systematic Reviews (Cochrane Library, CDSR),**

#1 vitamin d:ti,ab,kw (Word variations have been searched)

#2 Uterus Neoplasm:ti,ab,kw (Word variations have been searched)

#3 Endometrial Neoplasm:ti,ab,kw (Word variations have been searched)

#4 Uterine Cervical Neoplasm:ti,ab,kw (Word variations have been searched)

#5 Ovarian Neoplasm:ti,ab,kw (Word variations have been searched)

#6 Oviduct Neoplasm:ti,ab,kw (Word variations have been searched)

#7 Vaginal Neoplasm:ti,ab,kw (Word variations have been searched)

#8 Ulvar Neoplasm:ti,ab,kw (Word variations have been searched)

#9 #2 OR #3 OR #4 OR #5 OR #6 OR #7 OR #8

#10 #1 AND #9 (restricted as Cochrane Reviews or other reviews)

**NEWCASTLE - OTTAWA QUALITY ASSESSMENT SCALE**

**CASE CONTROL STUDIES**

Note: A study can be awarded a maximum of one star for each numbered item within the Selection and Exposure categories. A maximum of two stars can be given for Comparability.

**Selection**

1) Is the case definition adequate?

a) yes, with independent validation **🟑**

b) yes, eg record linkage or based on self reports

c) no description

2) Representativeness of the cases

a) consecutive or obviously representative series of cases **🟑**

b) potential for selection biases or not stated

3) Selection of Controls

a) community controls **🟑**

b) hospital controls

c) no description

4) Definition of Controls

a) no history of disease (endpoint) **🟑**

b) no description of source

**Comparability**

1) Comparability of cases and controls on the basis of the design or analysis

a) study controls for _______________ (Select the most important factor.) **🟑**

b) study controls for any additional factor **🟑** (This criteria could be modified to indicate specific control for a second important factor.)

**Exposure**

1) Ascertainment of exposure

a) secure record (eg surgical records) **🟑**

b) structured interview where blind to case/control status **🟑**

c) interview not blinded to case/control status

d) written self report or medical record only

e) no description

2) Same method of ascertainment for cases and controls

a) yes **🟑**

b) no

3) Non-Response rate

a) same rate for both groups **🟑**

b) non respondents described

c) rate different and no designation

| **Section/topic** | **#** | **Checklist item** | **Reported on page #** |
| --- | --- | --- | --- |
| **TITLE** | | |  |
| Title | 1 | Identify the report as a systematic review, meta-analysis, or both. | Page 1 (**Title**) |
| **ABSTRACT** | | |  |
| Structured summary | 2 | Provide a structured summary including, as applicable: background; objectives; data sources; study eligibility criteria, participants, and interventions; study appraisal and synthesis methods; results; limitations; conclusions and implications of key findings; systematic review registration number. | Page 2 (**ABSTRACT**) |
| **INTRODUCTION** | | |  |
| Rationale | 3 | Describe the rationale for the review in the context of what is already known. | Page 4，5 (**INTRODUCTION**) |
| Objectives | 4 | Provide an explicit statement of questions being addressed with reference to participants, interventions, comparisons, outcomes, and study design (PICOS). | Page 4, 5 (**INTRODUCTION**) |
| **METHODS** | | |  |
| Protocol and registration | 5 | Indicate if a review protocol exists, if and where it can be accessed (e.g., Web address), and, if available, provide registration information including registration number. | Page 6, 7 (**METHODS**) |
| Eligibility criteria | 6 | Specify study characteristics (e.g., PICOS, length of follow-up) and report characteristics (e.g., years considered, language, publication status) used as criteria for eligibility, giving rationale. | Page 6 (**Eligibility Criteria**) |
| Information sources | 7 | Describe all information sources (e.g., databases with dates of coverage, contact with study authors to identify additional studies) in the search and date last searched. | Page 6 (**Search strategy**) |
| Search | 8 | Present full electronic search strategy for at least one database, including any limits used, such that it could be repeated. | Page 6 (**Search strategy**) |
| Study selection | 9 | State the process for selecting studies (i.e., screening, eligibility, included in systematic review, and, if applicable, included in the meta-analysis). | Page 6, 7 (**Study Selection and Data Collection Process**) |
| Data collection process | 10 | Describe method of data extraction from reports (e.g., piloted forms, independently, in duplicate) and any processes for obtaining and confirming data from investigators. | Page 6, 7 (**Study Selection and Data Collection Process)** |
| Data items | 11 | List and define all variables for which data were sought (e.g., PICOS, funding sources) and any assumptions and simplifications made. | Page 6,7 (**Data Items**) |
| Risk of bias in individual studies | 12 | Describe methods used for assessing risk of bias of individual studies (including specification of whether this was done at the study or outcome level), and how this information is to be used in any data synthesis. | Page 6 (**Risk of Bias in Individual Studies**) |
| Summary measures | 13 | State the principal summary measures (e.g., risk ratio, difference in means). | Page 6, 7 (**Summary Measures and Planned Methods of Analysis**) |
| Synthesis of results | 14 | Describe the methods of handling data and combining results of studies, if done, including measures of consistency (e.g., I^2^) for each meta-analysis. | Page 6, 7 (**Summary Measures and Planned Methods of Analysis**) |

Page 1 of 2

| **Section/topic** | **#** | **Checklist item** | **Reported on page #** |
| --- | --- | --- | --- |
| Risk of bias across studies | 15 | Specify any assessment of risk of bias that may affect the cumulative evidence (e.g., publication bias, selective reporting within studies). | Page 7 (**Risk of Bias Across Studies**) |
| Additional analyses | 16 | Describe methods of additional analyses (e.g., sensitivity or subgroup analyses, meta-regression), if done, indicating which were pre-specified. | Page 7 (**Sensitivity or subgroup analyses**) |
| **RESULTS** | | |  |
| Study selection | 17 | Give numbers of studies screened, assessed for eligibility, and included in the review, with reasons for exclusions at each stage, ideally with a flow diagram. | Page 8 (**Study Selection,** Fig. 1) |
| Study characteristics | 18 | For each study, present characteristics for which data were extracted (e.g., study size, PICOS, follow-up period) and provide the citations. | Page 8 (**Study characteristics,** Table 1) |
| Risk of bias within studies | 19 | Present data on risk of bias of each study and, if available, any outcome level assessment (see item 12). | Page 8 (**Risk of Bias within Studies,** Table 2) |
| Results of individual studies | 20 | For all outcomes considered (benefits or harms), present, for each study: (a) simple summary data for each intervention group (b) effect estimates and confidence intervals, ideally with a forest plot. | Page 8, 9 (Table 1, Fig. 2, Fig. 3, Fig. 4, Fig. 5) |
| Synthesis of results | 21 | Present results of each meta-analysis done, including confidence intervals and measures of consistency. | Page 8, 9 (**Syntheses of Results,** Fig. 2, Fig. 3, Fig. 4, Fig. 5) |
| Risk of bias across studies | 22 | Present results of any assessment of risk of bias across studies (see Item 15). | Page 9 (**Risk of Bias Across Studies,** Fig. 6, Fig.7, Fig. 8, Fig. 9) |
| Additional analysis | 23 | Give results of additional analyses, if done (e.g., sensitivity or subgroup analyses, meta-regression [see Item 16]). | Page 8, 9 (**Sensitivity analysis and** **subgroup analysis**) |
| **DISCUSSION** | | |  |
| Summary of evidence | 24 | Summarize the main findings including the strength of evidence for each main outcome; consider their relevance to key groups (e.g., healthcare providers, users, and policy makers). | Page 10-12 (**Summary of Evidence**) |
| Limitations | 25 | Discuss limitations at study and outcome level (e.g., risk of bias), and at review-level (e.g., incomplete retrieval of identified research, reporting bias). | Page 12, 13 (**Limitations**) |
| Conclusions | 26 | Provide a general interpretation of the results in the context of other evidence, and implications for future research. | Page 13 (**Conclusion**) |
| **FUNDING** | | |  |
| Funding | 27 | Describe sources of funding for the systematic review and other support (e.g., supply of data); role of funders for the systematic review. | Page 14 (**Funding**) |

*From:*  Moher D, Liberati A, Tetzlaff J, Altman DG, The PRISMA Group (2009). Preferred Reporting Items for Systematic Reviews and Meta-Analyses: The PRISMA Statement. PLoS Med 6(7): e1000097. doi:10.1371/journal.pmed1000097

For more information, visit: **www.prisma-statement.org**.

Page 2 of 2

**Supplementary 3**  PRISMA 2009 checklist

| **Section/topic** | **#** | **Checklist item** | **Reported on page #** |
| --- | --- | --- | --- |
| **TITLE** | | |  |
| Title | 1 | Identify the report as a systematic review, meta-analysis, or both. | Page 1 (**Title**) |
| **ABSTRACT** | | |  |
| Structured summary | 2 | Provide a structured summary including, as applicable: background; objectives; data sources; study eligibility criteria, participants, and interventions; study appraisal and synthesis methods; results; limitations; conclusions and implications of key findings; systematic review registration number. | Page 2 (**ABSTRACT**) |
| **INTRODUCTION** | | |  |
| Rationale | 3 | Describe the rationale for the review in the context of what is already known. | Page 4，5 (**INTRODUCTION**) |
| Objectives | 4 | Provide an explicit statement of questions being addressed with reference to participants, interventions, comparisons, outcomes, and study design (PICOS). | Page 4, 5 (**INTRODUCTION**) |
| **METHODS** | | |  |
| Protocol and registration | 5 | Indicate if a review protocol exists, if and where it can be accessed (e.g., Web address), and, if available, provide registration information including registration number. | Page 6, 7 (**METHODS**) |
| Eligibility criteria | 6 | Specify study characteristics (e.g., PICOS, length of follow-up) and report characteristics (e.g., years considered, language, publication status) used as criteria for eligibility, giving rationale. | Page 6 (**Eligibility Criteria**) |
| Information sources | 7 | Describe all information sources (e.g., databases with dates of coverage, contact with study authors to identify additional studies) in the search and date last searched. | Page 6 (**Search strategy**) |
| Search | 8 | Present full electronic search strategy for at least one database, including any limits used, such that it could be repeated. | Page 6 (**Search strategy**) |
| Study selection | 9 | State the process for selecting studies (i.e., screening, eligibility, included in systematic review, and, if applicable, included in the meta-analysis). | Page 6, 7 (**Study Selection and Data Collection Process**) |
| Data collection process | 10 | Describe method of data extraction from reports (e.g., piloted forms, independently, in duplicate) and any processes for obtaining and confirming data from investigators. | Page 6, 7 (**Study Selection and Data Collection Process)** |
| Data items | 11 | List and define all variables for which data were sought (e.g., PICOS, funding sources) and any assumptions and simplifications made. | Page 6,7 (**Data Items**) |
| Risk of bias in individual studies | 12 | Describe methods used for assessing risk of bias of individual studies (including specification of whether this was done at the study or outcome level), and how this information is to be used in any data synthesis. | Page 6 (**Risk of Bias in Individual Studies**) |
| Summary measures | 13 | State the principal summary measures (e.g., risk ratio, difference in means). | Page 6, 7 (**Summary Measures and Planned Methods of Analysis**) |
| Synthesis of results | 14 | Describe the methods of handling data and combining results of studies, if done, including measures of consistency (e.g., I^2^) for each meta-analysis. | Page 6, 7 (**Summary Measures and Planned Methods of Analysis**) |

Page 1 of 2

| **Section/topic** | **#** | **Checklist item** | **Reported on page #** |
| --- | --- | --- | --- |
| Risk of bias across studies | 15 | Specify any assessment of risk of bias that may affect the cumulative evidence (e.g., publication bias, selective reporting within studies). | Page 7 (**Risk of Bias Across Studies**) |
| Additional analyses | 16 | Describe methods of additional analyses (e.g., sensitivity or subgroup analyses, meta-regression), if done, indicating which were pre-specified. | Page 7 (**Sensitivity or subgroup analyses**) |
| **RESULTS** | | |  |
| Study selection | 17 | Give numbers of studies screened, assessed for eligibility, and included in the review, with reasons for exclusions at each stage, ideally with a flow diagram. | Page 8 (**Study Selection,** Fig. 1) |
| Study characteristics | 18 | For each study, present characteristics for which data were extracted (e.g., study size, PICOS, follow-up period) and provide the citations. | Page 8 (**Study characteristics,** Table 1) |
| Risk of bias within studies | 19 | Present data on risk of bias of each study and, if available, any outcome level assessment (see item 12). | Page 8 (**Risk of Bias within Studies,** Table 2) |
| Results of individual studies | 20 | For all outcomes considered (benefits or harms), present, for each study: (a) simple summary data for each intervention group (b) effect estimates and confidence intervals, ideally with a forest plot. | Page 8, 9 (Table 1, Fig. 2, Fig. 3, Fig. 4, Fig. 5) |
| Synthesis of results | 21 | Present results of each meta-analysis done, including confidence intervals and measures of consistency. | Page 8, 9 (**Syntheses of Results,** Fig. 2, Fig. 3, Fig. 4, Fig. 5) |
| Risk of bias across studies | 22 | Present results of any assessment of risk of bias across studies (see Item 15). | Page 9 (**Risk of Bias Across Studies,** Fig. 6, Fig.7, Fig. 8, Fig. 9) |
| Additional analysis | 23 | Give results of additional analyses, if done (e.g., sensitivity or subgroup analyses, meta-regression [see Item 16]). | Page 8, 9 (**Sensitivity analysis and subgroup analysis**) |
| **DISCUSSION** | | |  |
| Summary of evidence | 24 | Summarize the main findings including the strength of evidence for each main outcome; consider their relevance to key groups (e.g., healthcare providers, users, and policy makers). | Page 10-12 (**Summary of Evidence**) |
| Limitations | 25 | Discuss limitations at study and outcome level (e.g., risk of bias), and at review-level (e.g., incomplete retrieval of identified research, reporting bias). | Page 12, 13 (**Limitations**) |
| Conclusions | 26 | Provide a general interpretation of the results in the context of other evidence, and implications for future research. | Page 13 (**Conclusion**) |
| **FUNDING** | | |  |
| Funding | 27 | Describe sources of funding for the systematic review and other support (e.g., supply of data); role of funders for the systematic review. | Page 14 (**Funding**) |

*From:*  Moher D, Liberati A, Tetzlaff J, Altman DG, The PRISMA Group (2009). Preferred Reporting Items for Systematic Reviews and Meta-Analyses: The PRISMA Statement. PLoS Med 6(7): e1000097. doi:10.1371/journal.pmed1000097

For more information, visit: **www.prisma-statement.org**.

Page 2 of 2

**Supplement 4** The detailed data of sensitivity analysis.

| Kick articles | ***I^2^*** | **P** | **OR** | **95% CI** |
| --- | --- | --- | --- | --- |
| Alessio Paffoni et al | 68.0% | 0.005 | 1.01 | 0.80, 1.28 |
| P Ingala et al | 67.8% | 0.005 | 1.09 | 0.87, 1.36 |
| Susanna D. Mitro et al | 33.6% | 0.172 | 1.15 | 0.96, 1.38 |
| Anne Zeleniuch-Jacquotte et al | 70.5% | 0.002 | 1.05 | 0.78, 1.40 |
| Fariba Almassinokiani et al | 69.9% | 0.003 | 1.03 | 0.82, 1.30 |
| Wei Zheng et al | 46.3% | 0.083 | 0.97 | 0.79, 1.19 |
| Alan A. Arslan et al | 70.1% | 0.003 | 1.08 | 0.84, 1.38 |
| Darius Salehin et al | 70.7% | 0.002 | 1.05 | 0.83, 1.31 |
